# Supplementary material for: Perspectives, benefits and challenges of a live OSCE during the COVID-19 pandemic in a cross-sectional study
Source: BMJ Open. 2022 Jun 3;12(6):e058845. doi: 10.1136/bmjopen-2021-058845 (PMC9170794; doi:10.1136/bmjopen-2021-058845)
Supplement: Author's manuscript [file bmjopen-2021-058845.draft_revisions.pdf]

BMJ Open is committed to open peer review. As part of this commitment we make the peer review history of every article we publish publicly available.

When an article is published we post the peer reviewers' comments and the authors' responses online. We also post the versions of the paper that were used during peer review. These are the versions that the peer review comments apply to.

The versions of the paper that follow are the versions that were submitted during the peer review process. They are not the versions of record or the final published versions. They should not be cited or distributed as the published version of this manuscript.

BMJ Open is an open access journal and the full, final, typeset and author-corrected version of record of the manuscript is available on our site with no access controls, subscription charges or pay-per-view fees (http://bmjopen.bmj.com).

If you have any questions on BMJ Open's open peer review process please email info.bmjopen@bmj.com

BMJ Open

An original article: Perspectives, benefits and challenges of a live OSCE during the COVID-19 Pandemic in a cross-sectional study

Journal:	*BMJ Open*
Manuscript ID	bmjopen-2021-058845
Article Type:	Original research
Date Submitted by the Author:	30-Oct-2021

Complete List of Authors:	Loda, Teresa; University Hospital Tuebingen, Department of Internal Medicine VI<br>Nevins, Andrew B; Stanford University School of Medicine, Department of Medicine<br>Erschens, Rebecca; University Medical Hospital Tuebingen, Internal Medicine, Department of Psychosomatic Medicine and Psychotherapy<br>Zipfel, Stephan; University Hospital Tübingen, Psychosomatic Medicine and Psychotherapy<br>Herrmann-Werner, Anne; University Hospital Tuebingen, Department for Psychsomatic Medicine and Psychotherapy

Keywords:	COVID-19, EDUCATION & TRAINING (see Medical Education & Training), MEDICAL EDUCATION & TRAINING

SCHOLARONE™
Manuscripts

I, the Submitting Author has the right to grant and does grant on behalf of all authors of the Work (as defined in the below author licence), an exclusive licence and/or a non-exclusive licence for contributions from authors who are: i) UK Crown employees; ii) where BMJ has agreed a CC-BY licence shall apply, and/or iii) in accordance with the terms applicable for US Federal Government officers or employees acting as part of their official duties; on a worldwide, perpetual, irrevocable, royalty-free basis to BMJ Publishing Group Ltd ("BMJ") its licensees and where the relevant Journal is co-owned by BMJ to the co-owners of the Journal, to publish the Work in this journal and any other BMJ products and to exploit all rights, as set out in our licence.

The Submitting Author accepts and understands that any supply made under these terms is made by BMJ to the Submitting Author unless you are acting as an employee on behalf of your employer or a postgraduate student of an affiliated institution which is paying any applicable article publishing charge ("APC") for Open Access articles. Where the Submitting Author wishes to make the Work available on an Open Access basis (and intends to pay the relevant APC), the terms of reuse of such Open Access shall be governed by a Creative Commons licence – details of these licences and which Creative Commons licence will apply to this Work are set out in our licence referred to above.

Other than as permitted in any relevant BMJ Author's Self Archiving Policies, I confirm this Work has not been accepted for publication elsewhere, is not being considered for publication elsewhere and does not duplicate material already published. I confirm all authors consent to publication of this Work and authorise the granting of this licence.

For peer review only

An original article: Perspectives, benefits and challenges of a live OSCE during the COVID-19 Pandemic in a cross-sectional study

Teresa Loda[1*], Rebecca Erschens[1], Andrew B Nevins[2], Stephan Zipfel[1,3], Anne Herrmann-Werner[1,4]

[1] Department of Internal Medicine VI/Psychosomatic Medicine and Psychotherapy, University Hospital Tuebingen, Tuebingen, Germany

[2]Department of Medicine, Division of Infectious Diseases, Stanford University School of Medicine, Stanford, CA, USA

[3] Faculty of Medicine, Eberhard-Karls University of Tuebingen, Tuebingen, Germany

[4] Competence Centre for University Teaching in Medicine, Baden-Wuerttemberg, Faculty of Medicine, University of Tuebingen, Tuebingen, Germany

Short title: OSCE in Covid-19

***Contact info (corresponding author):**

Dr. Teresa Loda, M. Sc.

Department of Psychosomatic Medicine and Psychotherapy, University Hospital Tuebingen, Osianderstr. 5, D-72076 Tuebingen, Germany

Phone: 0049 7071 29 73688

Fax: 0049 7071 294382

e-mail: teresa.loda@med.uni-tuebingen.de

For peer review only - http://bmjopen.bmj.com/site/about/guidelines.xhtml

Word count: 2794 words

ABSTRACT

Objectives

Restrictions due to the COVID-19 pandemic mandated fundamental changes to student evaluations, including the administration of the Observed Structured Clinical Exam (OSCE). This study aims to conduct an in-person OSCE to verify students' practical skills under necessary infection-control practices and the impact of face masks on student–patient interactions.

Design

This study presents a cross-sectional design.

Setting

The OSCE at the Medical School of Tuebingen takes place at the end of the third year of study.

Participants

A total of 149 students completed the examination as well as the survey (RR = 80.1%). All medical students were in their third year of study.

Primary and Secondary outcome measures

The aim of the OSCE is the examination of students' clinical and communication skills. Students, standardised patients (SP) and examiners completed a general evaluation of structure and content and a specific COVID-19 evaluation of hygienic measures, particularly face masks. Items were rated on a six-point Likert scale. Means, standard deviations, frequencies and percentages were calculated.

Results

149 students, 32 SPs and 59 examiners participated. The students rated the OSCE with 2.37 (±0.52) for preparation and 2.07 (±0.32) for content. They perceived the interaction to be

For peer review only - http://bmjopen.bmj.com/site/about/guidelines.xhtml

For peer review only

significantly disrupted by the use of face masks (3.03 ± 1.54) ($p < .001$) compared to the SPs (3.84±1.44) and the examiners (4.14 ± 1.55). In general, the three groups considered the use of face masking the OSCE to be helpful (1.60 ± 1.15).

Conclusions

An in-person OSCE, even in the midst of a global pandemic, is feasible and acceptable to both students and faculty. Although students felt less prepared than under non-pandemic circumstances, their performances on this OSCE was not lower.

For peer review only

Strengths and limitations of this study

- The study presents the evaluation of an in-person OSCE in the height of this pandemic
- Best to our knowledge, this is the first investigation of the influence of face masks regarding communication and interaction in an OSCE
- The perspective of all people involved in the OSCE were assessed.
- One limitation is that the students might feel more stressed during the OSCE as it is an examination
- The study was conducted at only one medical faculty

Key words: OSCE, COVID-19, undergraduate medical students, face mask.

For peer review only - http://bmjopen.bmj.com/site/about/guidelines.xhtml

INTRODUCTION

The COVID-19 pandemic resulted in significant adaptations for teaching in medical schools, with a forced emphasis on digitalisation; for example, e-learning platforms, virtual training and video-conferencing have become established strategies for teaching students and others studying in the medical field.[1-4] Initially, traditional face-to-face teaching formats such as physical examinations had to be paused completely due to stringent governmental orders and the need to develop appropriate infection-control practices.[5 6] This change in format had impacts on not only how to teach but also what to teach, as some clinical skills, in particular communication skills training, are difficult to adapt from in-person to remote sessions. However, telemedicine has become a valuable option for teaching such clinical and communication skills.[7]

Despite new opportunities, this alternative method of teaching remains challenging, particularly the question of how to perform corresponding, valid exams under restrictions required to prevent the spread of COVID-19. Various approaches had to be tried to accommodate different phases of the pandemic itself.[1 8] Catalogues of learning objectives also had to be adapted accordingly, resulting in direct impacts on the content of various exams. With great effort, exams themselves had to be altered, either reformulated as online versions with all the related data-protection implications or held in classrooms under strict hygienic measures. Clinical and communication skills examinations, in particular, were a highly significant challenge and were sometimes omitted entirely during the first COVID-19 semester in summer 2020 as the risk of infection was too high. This especially affected the creation and administration of observed, structured clinical examinations (OSCEs) with their interdisciplinary setting evaluated by teachers of various disciplines.[9] The OSCE represents a standard assessment tool for medical training and is considered the most reliable and valid clinical examination system in undergraduate training[10]; therefore, a way to implement the OSCE during the pandemic COVID-19 had to be identified. Several universities solved this problem by conducting virtual or online OSCEs using Zoom video-conferencing.[11-13] However, the degree of realism in the virtual OSCE is limited, and the OSCE's organizational team needed to be prepared to manage and resolve technical difficulties.[11]

For peer review only - http://bmjopen.bmj.com/site/about/guidelines.xhtml

When the curve of the COVID-19 pandemic flattened, the Medical Faculty of Tuebingen decided that courses including clinical examinations should go back to being held in person, maintaining appropriate and strict infection-control practices since such clinical encounters represent an essential component of medical training. This particularly affected the OSCE as the faculty's central interdisciplinary, clinical examination. In this context, appropriate hygiene measures were developed by the OSCE coordination team based on the university and government specifications in order to minimize infection risk for all persons involved in the OSCE including students, examiners, and SPs. Here, we describe not only our successes in this effort but also several of the challenges we faced.

Aim

This study aims to present one method for facilitating an OSCE during a real-world pandemic situation. Thus, the study investigated how this type of OSCE was evaluated by participating students in regard to preparation, content and difficulty as well as an authentic context. Furthermore, we investigated how the implemented hygiene measures influenced the OSCE, including the interaction and communication between students and SPs.

METHODS

Study design

This study presents a cross-sectional study at the University of Tuebingen.

The OSCE at the University of Tuebingen before the COVID-19 pandemic

The OSCE at the Medical School of Tuebingen takes place at the end of the third year of study. Nine clinical subjects participate in the OSCE: dermatology, general medicine, internal medicine, neurology, orthopaedics, paediatrics, psychosomatic medicine, radiology and surgery. The aim of the OSCE is the examination of students' clinical and communication skills. Clinical skills were represented by performing a clinical examination or by interpreting a medical report. Communication skills were assessed by the way the students interacted with the patients, e.g. by asking the patient for his or her name or introducing themselves using their name and function or by how they structured the communication with patients. Typically, the students are examined at 16 exam stations representing basic tasks such as

For peer review only - http://bmjopen.bmj.com/site/about/guidelines.xhtml

taking a patient's medical history, performing a clinical examination or interpreting medical findings (e.g. an x-ray). The task at each station lasts six minutes with a one-minute break to change places. All interactive stations (medical history, physical examination) include standardized patients (SPs) to provide a more realistic experience Students are evaluated by discipline-specific examiners using an electronic standardised checklist consisting of 25 items on a tablet. Usually, about 180 medical students take part in the OSCE per semester.

Implementation of the OSCE at the University of Tuebingen during the COVID-19 pandemic

During the pandemic, OSCEs took place in October 2020. The 7-day incidence rate at this time was 88 per 100,000 (https://www.tagblatt.de/Nachrichten/Live-Blog, 11.05.21). Rapid testing had not yet been established as a means to test all participants for the coronavirus. Thus, the challenge was to adapt the OSCE to the university's hygiene measures including maintaining a social distance of at least 1.5 meters during the OSCE and limiting the number of persons involved. As the number of participating students per run was limited to 16, the OSCE was extended to three instead of two days, including six thematic blocks. The general structure of nine clinical subjects and 16 exam stations was retained. In addition, the exam time was kept to six minutes per station. However, the break-time between stations was doubled to two minutes to accommodate performing hygienic procedures (e.g. wiping all surfaces). All persons involved in the OSCE, including students, examiners, SPs and the coordination team, were required to wear face masks (minimum standard FFP1) for the total duration of the OSCE. This also meant that several established stations had to be adapted because, as a result, several procedures were not possible (e.g. examination of the larynx, facial nerve testing). For physical examinations, students were required to wear surgical gloves, dispose of them after each examination, and put on a new pair for each new SP they met.

Evaluation

The evaluation of the OSCE during the COVID-19 pandemic consisted of two parts: (1) a general evaluation (established standard) and (2) a COVID-19 pandemic-specific evaluation (resulting from changes due to COVID-19).

For peer review only - http://bmjopen.bmj.com/site/about/guidelines.xhtml

General evaluation

The general evaluation included questions on the organization of the OSCE as well as rating the preparation, content and difficulty of each exam station. The students were also asked if they felt confident in their ability to perform each examined task in a real-word situation. All items were assessed by academic grades (1–6). Finally, students were given the opportunity to comment on the OSCE in a free-text section.

The COVID-19 pandemic-specific evaluation

The specific evaluation in relation to COVID-19 referred to the effects of wearing face masks and was divided into the following topics: *interaction, non-verbal communication, face mask in general,* and *face mask during OSCE*. An example of an item for *interaction* was 'The face mask influenced the interaction with the SP'. *Non-verbal communication* was assessed by the item 'They [students] made more use of the eyes in order to communicate with the SP'. An example of an item for *face mask in general* was represented by 'In general, I consider wearing a face mask as reasonable'. The topic *face mask during OSCE* was measured by items such as: 'Wearing a face mask affects taking the medical history/performing a physical examination'. All items were rated on a six-point Likert scale ranging from 1 ('completely') to 6 ('not at all').

Procedure

The participating students first completed the OSCE and then completed the general and COVID-19-specific evaluations. In addition, SPs and examiners completed the general evaluation after the OSCE. Both groups rated the students' clinical skills, communication skills and empathy after completing each student's checklist in the two-minute-break window.

Ethics

The study received ethics approval from the Ethics Committee of Tuebingen Medical Faculty (no. 314/2020BO2). Participation was voluntary, and students were not reimbursed or rewarded in any way for participating. All participants provided their written informed consent, and all of their responses and data were kept anonymous.

For peer review only

Patient and Public Involvement

Patients or the public were not involved in the design, or conduct, or reporting, or dissemination plans of our research.

Data analysis

A sample size of 90 students were needed for analysis. The normal distribution of the data was confirmed by using the Kolmogorov–Smirnov test. Descriptive data, including mean values *(M),* standard deviations *(SD),* frequencies and percentages of relevant factors, were calculated. Any missing value was replaced with the mean value. *T* test for independent samples and ANOVAs were conducted to compare the results between the three groups including students, SPs and examiners. When data were not normally distributed, the Kruskal-Wallis test was performed to compare results. Bonferroni correction was used to correct multiple testing.[14] The Statistical Package for the Social Sciences version 26.0 (IBM, Armonk, NY, USA) was used for data analysis. The level of significance was set at $p < .05$.

RESULTS

Demographics

A total of 149 students completed the examination as well as the survey (RR = 80.1%). Also participating in the survey were 32 standardized patients (RR = 91.4%) and 59 examiners (RR = 92.2%). All medical students were in their third year of study.

General evaluation

Students evaluated the OSCE in regard to preparation at 2.37 (±0.52) and content and difficulty at 2.07 (±0.32). The confidence to perform the tasks in a real-world setting was rated 2.36 (±0.47). Both SPs and examiners rated the students' clinical skills as good (SPs: 1.83±0.79; examiners: 1.81±0.72, p > .05). Similar results were found for students' communication skills (SPs: 1.72±0.77; examiners: 1.70±0.57, p > .05). In regard to students' empathy with SPs, there was a significant effect (p < .05) showing a higher rating by the examiners (1.51±0.55) when compared to the SPs (1.79±0.78).

COVID-19-specific evaluation

For peer review only - http://bmjopen.bmj.com/site/about/guidelines.xhtml

Interaction and non-verbal communication

In regard to interaction and non-verbal communication, the results showed significant differences (p < .001) for the three groups, i.e. students, SPs and examiners (see Table 1 and Figure 1). Students rated the single items of interaction and non-verbal communication with higher agreement than did the SPs and examiners. The students were significantly in greater agreement that wearing a face mask could lead to misunderstandings in comparison to the SPs and examiners, who both disagreed with the statement. Overall, compared to students and examiners, the SPs were in significant disagreement that face masks affected non-verbal communication by, e.g. speaking more loudly or using more gestures.

Table 1. *Interaction and non-verbal communication.*

**Items***	**Students**		**SPs**		**Examiners**		**Statistics**
	M	SD	M	SD	M	SD	p-value
In general, interaction is affected by face masks.	3.03	1.54	3.84	1.44	4.14	1.55	< .001
Face masks have a negative impact on specific interactions with SPs.	2.92	1.58	3.59	1.58	3.85	1.68	< .001
Face masks lead to misunderstandings with SPs.	3.85	1.59	5.43	1.04	4.82	1.38	< .001

Figure 1 about here

The use of face masks in general and in the OSCE

Students, SPs and examiners disagreed that wearing face masks had a negative impact on their well-being (range: 4.19–4.91). They all rated the face mask as useful in the OSCE (range: 1.52–1.79). However, students were more likely to agree that they would have preferred the option of participating in the OSCE the following semester without wearing a face mask (3.03±1.84). In regard to the content of the examination, SPs and examiners significantly disagreed that wearing a mask interfered with conducting an anamnesis. Furthermore, the SPs significantly disagreed that wearing a face mask interfered with performing a physical examination during

For peer review only - http://bmjopen.bmj.com/site/about/guidelines.xhtml

the OSCE. In addition, compared to SPs and examiners, students significantly disagreed that face masks affected communication during the OSCE. SPs and examiners significantly disagreed that face masks have negative impact on the students' grade. Please see Table 2 and Figure 2 for additional details.

Table 2. *The use of face masks in general and in the OSCE.*

Items*	Students		SP		Examiner		Statistics
	M	SD	M	SD	M	SD	p-value
Face masks, in general, are useful.	1.58	1.08	2.67	2.13	1.92	1.34	< .001
A face mask has a negative impact on my well-being.	4.19	1.76	4.91	1.35	4.54	1.56	> .05
Wearing a face mask causes physical symptoms (e.g. headaches, getting less air, etc.).	4.68	1.63	5.47	0.95	4.79	1.68	< .05
Wearing a face mask interfered with conducting the anamnesis.	3.68	1.73	4.70	1.34	4.76	1.56	< .001
Wearing a face mask interfered with performing a physical examination during the OSCE.	3.81	1.65	5.33	1.04	4.44	1.80	< .001
Face masks have a negative impact on the academic grade.	4.03	1.64	4.93	1.86	5.00	1.58	< .001

*Items presented are from the students' questionnaire version; SPs/examiners had respective corresponding items (e.g. 'Wearing face masks interfered with the students conducting the anamnesis'.

Figure 2 about here

DISCUSSION

This study investigated the implementation of an OSCE during a pandemic. Regarding students' clinical and communication skills, SPs and examiners rated them as good, while the students themselves felt less confident about performing the required tasks in a real-world

For peer review only - http://bmjopen.bmj.com/site/about/guidelines.xhtml

setting. When focussing on the changes required for appropriate hygienic measures, the SPs reported that face masks affected non-verbal communication, including speaking louder or using more eye contact, less in comparison to the examiners and students. Students, however, rated the face masks as having influenced the interaction and non-verbal communication with the SPs; therefore, they used more eye contact and spoke louder. In general, all participants found face masks to be a useful tool in the OSCE during the pandemic and reported that the masks did not interfere with performing the OSCE in general.

Evaluation of an OSCE during a pandemic

When compared to previous results, i.e. OSCE before COVID-19, students reported that they felt less prepared for this OSCE.[9] They also evaluated the content as more challenging and the difficulty as greater for the OSCE under the conditions required due to the pandemic.[9] Furthermore, they felt less confident about performing tasks such as taking a medical history or conducting a physical examination in a real-world setting, although the SPs and examiners rated their clinical and communications skills as good. The SPs' and examiners ratings of clinical and communication skills were similar to those of students' exam performances on previous OSCEs.[15 16] The reason may be that problematic circumstances during the semester (digital teaching, less emphasis on clinical skills) seem to have been counterbalanced by more-lenient examiners who kept these difficulties in mind.

Furthermore, the OSCE presents a stressful exam situation in which many students feel distressed and anxious regardless of unusual circumstances such as those caused by the COVID-19 pandemic.[9 17 18] This year, the students experienced even greater distress during the OSCE since the academic courses to prepare them for it were transferred to online formats. Thus, students might feel less confident about performing the tasks in a real-world situation.

Face masks and interaction

In regard to interaction and non-verbal interaction, students were afraid that wearing face masks might affect their interaction with the SPs and that non-verbal communication might interfere with the SP during the tasks. Similar fears were also reported by psychiatrists who emphasised that the face masks contribute to a psycho-social barrier in the patient–physician interaction.[19] In addition, students agreed to speak louder when wearing a face mask. This phenomenon was confirmed by a previous study where a speech intelligibility assessment of

For peer review only - http://bmjopen.bmj.com/site/about/guidelines.xhtml

protective face masks and air-purifying respirators was investigated.[20] However, examiners and SPs perceived the face masks as less disruptive than the students did. Examiners as clinicians may have become accustomed to them. Furthermore, SPs were specifically instructed in advance about how to communicate while wearing a face mask. Finally, all participants agreed that wearing face masks did not lead to any misunderstandings with the SPs, which was in contrast to previous studies that investigated face masks in relation to patient–physician communication.[21]

Face masks in the OSCE

Wearing face masks was seen as useful in general, as well as in the OSCE exam, by students, SPs and examiners. This finding aligns with those of previous investigations of face masks.[21-23] Moreover, all participants disagreed that face masks might interfere with performing a physical examination or taking a medical history during the OSCE. While Campagne [24] emphasised that face masks contribute to distress in patient communication, the students in the present study reported that they did not affect the communication in the OSCE and that they were able to structure and take charge of the conversation despite face masks.[24] SPs and examiners agreed with the students. Medical students are well-informed about COVID-19 and are aware of its consequences in academic and healthcare contexts.[25 26] Thus, the students might have felt confident about conducting a conversation while wearing a face mask in the OSCE. All participants also agreed that wearing face masks had no negative impact on the students' grades in the OSCE.

Strengths and limitations

Based on the results, a strength of this study includes the finding that an in-person OSCE can be effectively performed even during a severe pandemic if it is done in a thoughtful and structured format, keeping both the educational objectives as well as proper infection-control practices in mind. Moreover, best to our knowledge, the study is the first investigation of the influence of face masks regarding communication and interaction in an OSCE. Furthermore, the perspective of all people involved in the OSCE were assessed.

A limitation of this study is the higher level of stress in medical students during the OSCE as it is an examination. Additionally, the study was conducted at only one medical faculty; thus, the results cannot be generalized. Despite its limitations, we believe that this study

For peer review only - http://bmjopen.bmj.com/site/about/guidelines.xhtml

contributes to the knowledge about how to conduct effective OSCEs and possibly other student assessments in unusual circumstances such as the COVID-19 pandemic, as well as the challenges that must be considered.

Conclusion

Wearing face masks did not interfere with student performance regarding clinical and communication skills in the OSCE exam. Students felt less prepared for the OSCE as 'refresher' courses were missing before the OSCE took place, and academic courses were transferred to online formats where relevant teaching elements, e.g. of physical examination, could be lost.[25] Again, these results showed that students were aware of the circumstances and consequences of COVID-19. Future research might also focus on the students' grades and performances in the OSCE during the pandemic, including a comparison with pre-pandemic OSCE results before the restrictions and changes required to prevent the spread of COVID-19.[9]

Acknowledgements
We thank Lea Herschbach for her support as study assistant.

Declaration of interest statement
The authors declare that they have no competing interests.

Funding/Support
We acknowledge support with financing publication fees by 'Deutsche Forschungsgemeinschaft' and Open Access Publishing Fund of the University of Tuebingen (both grant numbers are not applicable).

Data availability statement
Complete data are available on reasonable request to the corresponding author.

Notes on contributors

Teresa Loda, Dr, M Sc, is a psychologist and research scientist at the Department for Psychosomatic Medicine and Psychotherapy at University Hospital Tübingen.

Andrew B. Nevins, Assitant Prof, MD, is a clinical assistant professor of medicine (infectious diseases) at the Standford University School of Medicine.

For peer review only - http://bmjopen.bmj.com/site/about/guidelines.xhtml

Rebecca Erschens, Dr, M Sc is a clinical psychologist, research scientist and lecturer at the Department for Psychosomatic Medicine and Psychotherapy at University Medical Hospital Tuebingen.

Stephan Zipfel Prof., MD., is Professor for Psychosomatic Medicine and Psychotherapy at University Hospital Tuebingen, and dean for student affairs at the Faculty of Medicine of Tuebingen University.

Anne Herrmann-Werner, Prof, MD, MME, a physician for psychosomatic medicine and psychotherapy by training, is full professor for Medical Education at the University Hospital and Medical Faculty Tübingen.

Author's contribution

AHW and TL were responsible for the design and conduction the study, as well as acquisition, analysis and interpretation of data. AHW and TL drafted the first version of the manuscript. RE and AN was involved in data analyses and interpretation and revised the manuscript critically. SZ made substantial contributions to the study design and revised the manuscript critically. All authors approved the final version of the manuscript and agreed to be accountable for all aspects of the work.

Figure caption

Figure 1. Face mask and non-verbal communication in patient–physician communication. Items presented are from the students' questionnaire version; SPs/examiners had respective corresponding items (e.g. 'the student spoke louder'). All items were rated on a six-point Likert scale ranging from 1 ('completely') to 6 ('not at all').

Figure 2. Face mask and patient-physician communication in the OSCE. Items presented are from the students' questionnaire version; SPs/examiners had respective corresponding items (e.g. 'the student could structure the conversation despite face mask'). All items were rated on a six-point Likert scale from ranging from 1 ('completely') to 6 ('not at all').

References

1. Rose S. Medical Student Education in the Time of COVID-19. *Jama* 2020;323(21):2131-32. doi: 10.1001/jama.2020.5227 [published Online First: 2020/04/02]
2. Liang ZC, Ooi SBS, Wang W. Pandemics and Their Impact on Medical Training: Lessons From Singapore. *Academic medicine : journal of the Association of American Medical Colleges* 2020;95(9):1359-61. doi: 10.1097/ACM.0000000000003441

For peer review only - http://bmjopen.bmj.com/site/about/guidelines.xhtml

3. Evans DJ, Bay BH, Wilson TD, et al. Going virtual to support anatomy education: A STOPGAP in the midst of the Covid-19 pandemic: Wiley Online Library, 2020.
4. Sandars J, Correia R, Dankbaar M, et al. Twelve tips for rapidly migrating to online learning during the COVID-19 pandemic. *MedEdPublish* 2020;9
5. Whelan A PJ, Young G, Catanese V, McKinney R. . Interim guidance on medical students' participation in direct patient contact activities: principles and guidelines. *Association of American Medical Colleges* 2020
6. Whelan A PJ, Young. Guidance on medical stu-dents' clinical participation: effective immediatel. *Academic Medicine* 2020
7. Iancu AM, Kemp MT, Alam HB. Unmuting Medical Students' Education: Utilizing Telemedicine During the COVID-19 Pandemic and Beyond. *J Med Internet Res* 2020;22(7):e19667. doi: 10.2196/19667
8. Theoret C, Ming X. Our education, our concerns: The impact on medical student education of COVID-19. *Medical education* 2020;54(7):591-92. doi: 10.1111/medu.14181 [published Online First: 2020/05/23]
9. Graf J, Smolka R, Holderried F, et al. Ten years of objective structured clinical examination at the medical Faculty of Tübingen, Germany: item analysis and students' satisfaction. *J Health Sci Educ* 2018;2(2):133.
10. Walters K, Osborn D, Raven P. The development, validity and reliability of a multimodality objective structured clinical examination in psychiatry. *Medical Education* 2005;39(3):292-98. doi: https://doi.org/10.1111/j.1365-2929.2005.02091.x
11. Khan KZ, Ramachandran S, Gaunt K, et al. The Objective Structured Clinical Examination (OSCE): AMEE Guide No. 81. Part I: an historical and theoretical perspective. *Med Teach* 2013;35(9):e1437-46. doi: 10.3109/0142159x.2013.818634 [published Online First: 2013/08/24]
12. Kakadia R, Chen E, Ohyama H. Implementing an online OSCE during the COVID-19 pandemic. *Journal of dental education* 2020
13. Donn J, Scott JA, Binnie V, et al. A pilot of a Virtual Objective Structured Clinical Examination in dental education. A response to COVID-19. *European Journal of Dental Education* 2020
14. Fahrmeir L, Heumann C, Künstler R, et al. Statistik: Der weg zur datenanalyse: Springer-Verlag 2016.
15. Graf J, Loda T, Zipfel S, et al. Communication skills of medical students: survey of self- and external perception in a longitudinally based trend study. *BMC Medical Education* 2020;20(1):149. doi: 10.1186/s12909-020-02049-w
16. Graf J, Smolka R, Simoes E, et al. Communication skills of medical students during the OSCE: Gender-specific differences in a longitudinal trend study. *BMC Medical Education* 2017;17(1):75. doi: 10.1186/s12909-017-0913-4
17. Brand HS, Schoonheim-Klein M. Is the OSCE more stressful? Examination anxiety and its consequences in different assessment methods in dental education. *European Journal of Dental Education* 2009;13(3):147-53. doi: https://doi.org/10.1111/j.1600-0579.2008.00554.x
18. Gilani S, Pankhania K, Aruketty M, et al. Twelve tips to organise a mock OSCE. *Medical Teacher* 2021:1-20. doi: 10.1080/0142159X.2021.1887465
19. Padhy SK, Rina K, Sarkar S. Smile, grimace or grin? Recalibrating psychiatrist-patient interaction in the era of face masks. *Asian journal of psychiatry* 2020;53:102389-89. doi: 10.1016/j.ajp.2020.102389 [published Online First: 2020/08/27]
20. Palmiero AJ, Symons D, Morgan JW, et al. Speech intelligibility assessment of protective facemasks and air-purifying respirators. *Journal of Occupational and Environmental Hygiene* 2016;13(12):960-68. doi: 10.1080/15459624.2016.1200723
21. Marler H, Ditton A. "I'm smiling back at you": Exploring the impact of mask wearing on communication in healthcare. *International Journal of Language & Communication Disorders* 2021;56(1):205-14. doi: https://doi.org/10.1111/1460-6984.12578
22. Spitzer M. Masked education? The benefits and burdens of wearing face masks in schools during the current Corona pandemic. *Trends in Neuroscience and Education* 2020;20:100138. doi: https://doi.org/10.1016/j.tine.2020.100138

For peer review only - http://bmjopen.bmj.com/site/about/guidelines.xhtml

23. Bakhit M, Krzyzaniak N, Scott AM, et al. Downsides of face masks and possible mitigation strategies: a systematic review and meta-analysis. *BMJ Open* 2021;11(2):e044364. doi: 10.1136/bmjopen-2020-044364
24. Campagne DM. The problem with communication stress from face masks. *Journal of Affective Disorders Reports* 2021;3:100069. doi: https://doi.org/10.1016/j.jadr.2020.100069
25. Loda T, Löffler T, Erschens R, et al. Medical education in times of COVID-19: German students' expectations – A cross-sectional study. *PLOS ONE* 2020;15(11):e0241660. doi: 10.1371/journal.pone.0241660
26. Taghrir MH, Borazjani R, Shiraly R. COVID-19 and Iranian Medical Students; A Survey on Their Related-Knowledge, Preventive Behaviors and Risk Perception. *Arch Iran Med* 2020;23(4):249-54. doi: 10.34172/aim.2020.06

For peer review only

For peer review only - http://bmjopen.bmj.com/site/about/guidelines.xhtml

Figure 1

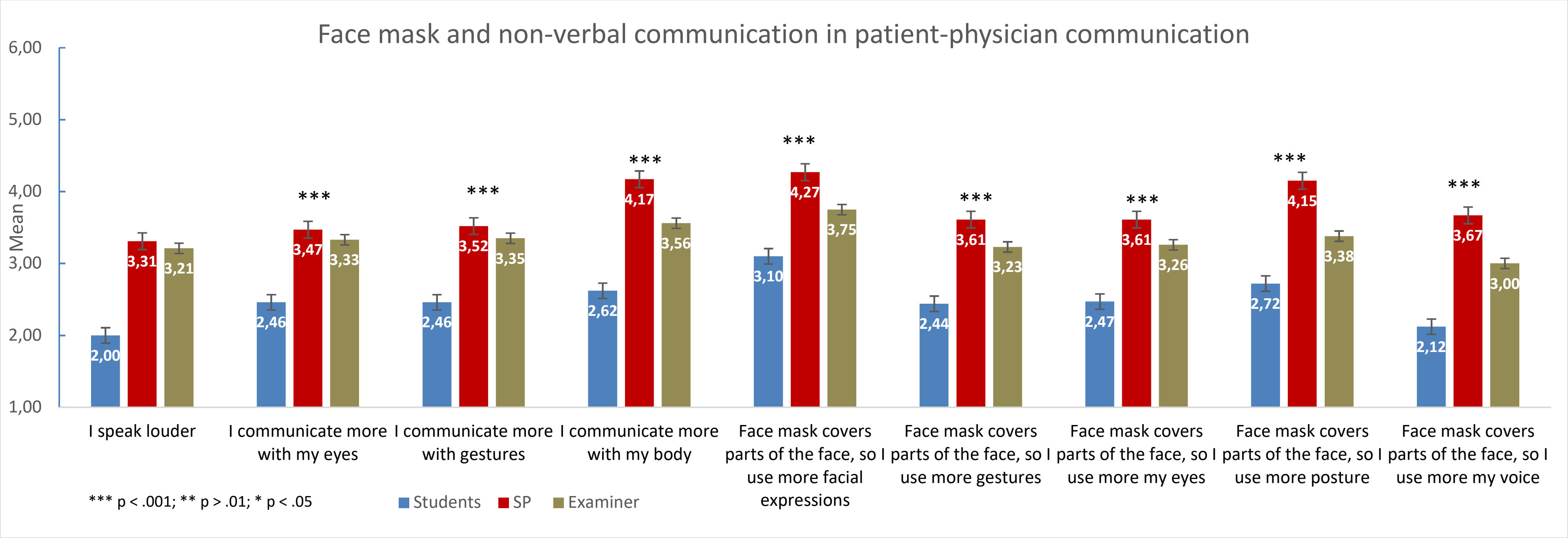
Face mask and non-verbal communication in patient-physician communication
6,00
5,00
4,00
3,00
2,00
1,00
Mean
3,31
3,21
2,00
***
3,47
3,33
2,46
***
3,52
3,35
2,46
***
4,17
3,56
2,62
***
4,27
3,75
3,10
***
3,61
3,23
2,44
***
3,61
3,26
2,47
***
4,15
3,38
2,72
***
3,67
3,00
2,12
I speak louder
I communicate more with my eyes
I communicate more with gestures
I communicate more with my body
Face mask covers parts of the face, so I use more facial expressions
Face mask covers parts of the face, so I use more gestures
Face mask covers parts of the face, so I use more my eyes
Face mask covers parts of the face, so I use more posture
Face mask covers parts of the face, so I use more my voice
*** p < .001; ** p > .01; * p < .05
Students
SP
Examiner

Figure 2

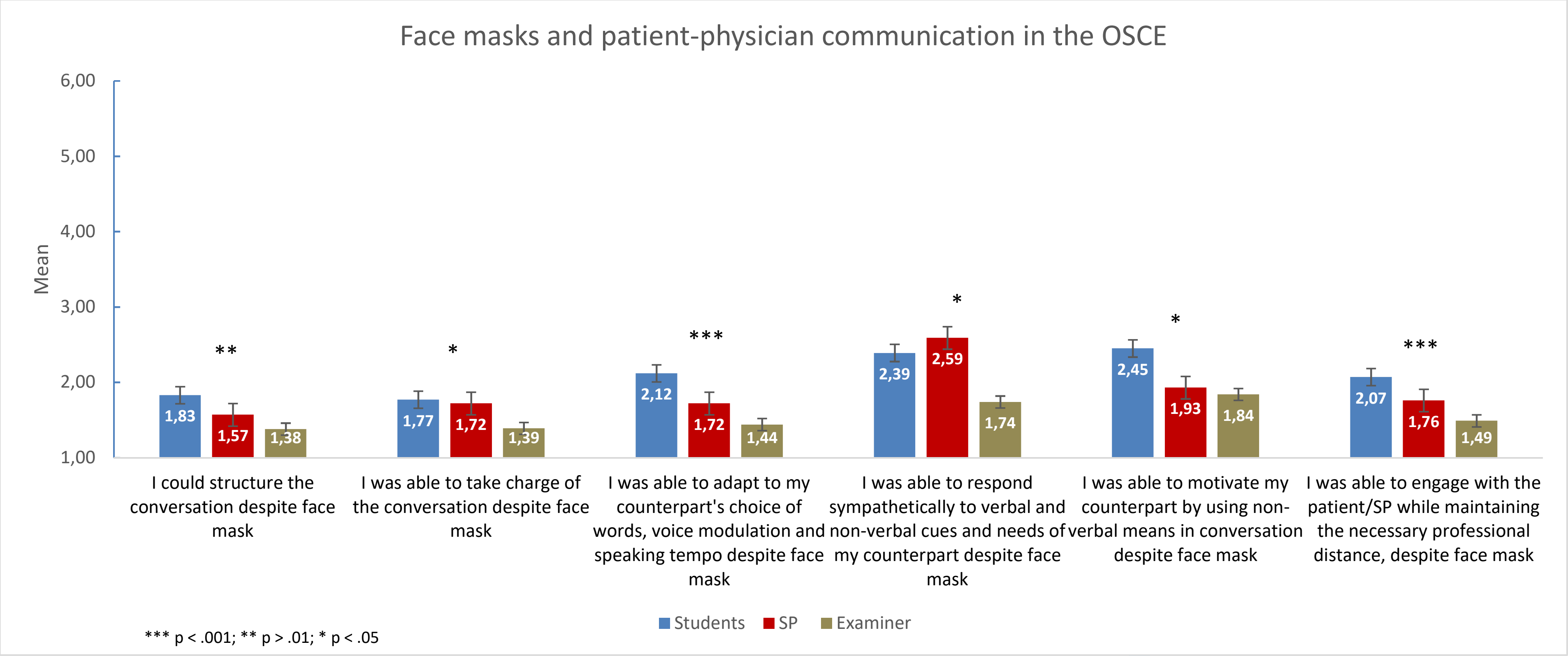
Face masks and patient-physician communication in the OSCE
Mean
6,00
5,00
4,00
3,00
2,00
1,00
**
*
***
*
*
***
1,83
1,57
1,38
1,77
1,72
1,39
2,12
1,72
1,44
2,39
2,59
1,74
2,45
1,93
1,84
2,07
1,76
1,49
I could structure the conversation despite face mask
I was able to take charge of the conversation despite face mask
I was able to adapt to my counterpart's choice of words, voice modulation and speaking tempo despite face mask
I was able to respond sympathetically to verbal and non-verbal cues and needs of my counterpart despite face mask
I was able to motivate my counterpart by using non-verbal means in conversation despite face mask
I was able to engage with the patient/SP while maintaining the necessary professional distance, despite face mask
Students
SP
Examiner
*** p < .001; ** p > .01; * p < .05

For peer review only

STROBE 2007 (v4) Statement—Checklist of items that should be included in reports of *cross-sectional studies*

Section/Topic	Item #	Recommendation	Reported on page #
**Title and abstract**	1	(*a*) Indicate the study's design with a commonly used term in the title or the abstract	1
		(*b*) Provide in the abstract an informative and balanced summary of what was done and what was found	2-3
**Introduction**			**4-6**
Background/rationale	2	Explain the scientific background and rationale for the investigation being reported	4-5
Objectives	3	State specific objectives, including any prespecified hypotheses	5
**Methods**			**5-8**
Study design	4	Present key elements of study design early in the paper	5
Setting	5	Describe the setting, locations, and relevant dates, including periods of recruitment, exposure, follow-up, and data collection	6
Participants	6	(*a*) Give the eligibility criteria, and the sources and methods of selection of participants	5-6
Variables	7	Clearly define all outcomes, exposures, predictors, potential confounders, and effect modifiers. Give diagnostic criteria, if applicable	6-7
Data sources/ measurement	8*	For each variable of interest, give sources of data and details of methods of assessment (measurement). Describe comparability of assessment methods if there is more than one group	6-7
Bias	9	Describe any efforts to address potential sources of bias	*N.A*
Study size	10	Explain how the study size was arrived at	8
Quantitative variables	11	Explain how quantitative variables were handled in the analyses. If applicable, describe which groupings were chosen and why	8
Statistical methods	12	(*a*) Describe all statistical methods, including those used to control for confounding	8
		(*b*) Describe any methods used to examine subgroups and interactions	8
		(*c*) Explain how missing data were addressed	8
		(*d*) If applicable, describe analytical methods taking account of sampling strategy	n.a.
		(*e*) Describe any sensitivity analyses	n.a.
**Results**			8-10

For peer review only

Participants	13*	(a) Report numbers of individuals at each stage of study—eg numbers potentially eligible, examined for eligibility, confirmed eligible, included in the study, completing follow-up, and analysed	8
		(b) Give reasons for non-participation at each stage	n.a.
		(c) Consider use of a flow diagram	n.a.
Descriptive data	14*	(a) Give characteristics of study participants (eg demographic, clinical, social) and information on exposures and potential confounders	8
		(b) Indicate number of participants with missing data for each variable of interest	8
Outcome data	15*	Report numbers of outcome events or summary measures	8-9
Main results	16	(*a*) Give unadjusted estimates and, if applicable, confounder-adjusted estimates and their precision (eg, 95% confidence interval). Make clear which confounders were adjusted for and why they were included	9-10
		(*b*) Report category boundaries when continuous variables were categorized	9-10
		(*c*) If relevant, consider translating estimates of relative risk into absolute risk for a meaningful time period	9-10
Other analyses	17	Report other analyses done—eg analyses of subgroups and interactions, and sensitivity analyses	n.a.
**Discussion**			10-13
Key results	18	Summarise key results with reference to study objectives	10-12
Limitations	19	Discuss limitations of the study, taking into account sources of potential bias or imprecision. Discuss both direction and magnitude of any potential bias	12
Interpretation	20	Give a cautious overall interpretation of results considering objectives, limitations, multiplicity of analyses, results from similar studies, and other relevant evidence	11-13
Generalisability	21	Discuss the generalisability (external validity) of the study results	13
**Other information**			13
Funding	22	Give the source of funding and the role of the funders for the present study and, if applicable, for the original study on which the present article is based	13

*Give information separately for cases and controls in case-control studies and, if applicable, for exposed and unexposed groups in cohort and cross-sectional studies.

Note: An Explanation and Elaboration article discusses each checklist item and gives methodological background and published examples of transparent reporting. The STROBE checklist is best used in conjunction with this article (freely available on the Web sites of PLoS Medicine at http://www.plosmedicine.org/, Annals of Internal Medicine at http://www.annals.org/, and Epidemiology at http://www.epidem.com/). Information on the STROBE Initiative is available at www.strobe-statement.org.

For peer review only

An original article: Perspectives, benefits and challenges of a live OSCE during the COVID-19 Pandemic in a cross-sectional study

Journal:	*BMJ Open*
Manuscript ID	bmjopen-2021-058845.R1
Article Type:	Original research
Date Submitted by the Author:	02-Mar-2022
Complete List of Authors:	Loda, Teresa; University Hospital Tubingen Department of Internal Medicine, Department for Psychosomatic Medicine and Psychotherapy<br>Erschens, Rebecca; University Hospital Tubingen Department of Internal Medicine, Department of Psychosomatic Medicine and Psychotherapy<br>Nevins, Andrew B; Stanford University School of Medicine, Department of Medicine<br>Zipfel, Stephan; University Hospital Tubingen Department of Internal Medicine, Psychosomatic Medicine and Psychotherapy<br>Herrmann-Werner, Anne; University Hospital Tubingen Department of Internal Medicine, Department for Psychsomatic Medicine and Psychotherapy
**Primary Subject Heading**:	Medical education and training
Secondary Subject Heading:	Communication
Keywords:	COVID-19, EDUCATION & TRAINING (see Medical Education & Training), MEDICAL EDUCATION & TRAINING

I, the Submitting Author has the right to grant and does grant on behalf of all authors of the Work (as defined in the below author licence), an exclusive licence and/or a non-exclusive licence for contributions from authors who are: i) UK Crown employees; ii) where BMJ has agreed a CC-BY licence shall apply, and/or iii) in accordance with the terms applicable for US Federal Government officers or employees acting as part of their official duties; on a worldwide, perpetual, irrevocable, royalty-free basis to BMJ Publishing Group Ltd ("BMJ") its licensees and where the relevant Journal is co-owned by BMJ to the co-owners of the Journal, to publish the Work in this journal and any other BMJ products and to exploit all rights, as set out in our licence.

The Submitting Author accepts and understands that any supply made under these terms is made by BMJ to the Submitting Author unless you are acting as an employee on behalf of your employer or a postgraduate student of an affiliated institution which is paying any applicable article publishing charge ("APC") for Open Access articles. Where the Submitting Author wishes to make the Work available on an Open Access basis (and intends to pay the relevant APC), the terms of reuse of such Open Access shall be governed by a Creative Commons licence – details of these licences and which Creative Commons licence will apply to this Work are set out in our licence referred to above.

Other than as permitted in any relevant BMJ Author's Self Archiving Policies, I confirm this Work has not been accepted for publication elsewhere, is not being considered for publication elsewhere and does not duplicate material already published. I confirm all authors consent to publication of this Work and authorise the granting of this licence.

For peer review only

An original article: Perspectives, benefits and challenges of a live OSCE during the COVID-19 Pandemic in a cross-sectional study

Teresa Loda[1*], Rebecca Erschens[1], Andrew B Nevins[2], Stephan Zipfel[1,3], Anne Herrmann-Werner[1,4]

[1] Department of Internal Medicine VI/Psychosomatic Medicine and Psychotherapy, University Hospital Tuebingen, Tuebingen, Germany

[2]Department of Medicine, Division of Infectious Diseases, Stanford University School of Medicine, Stanford, CA, USA

[3] Faculty of Medicine, Eberhard-Karls University of Tuebingen, Tuebingen, Germany

[4] Competence Centre for University Teaching in Medicine, Baden-Wuerttemberg, Faculty of Medicine, University of Tuebingen, Tuebingen, Germany

Short title: OSCE in Covid-19

***Contact info (corresponding author):**

Dr. Teresa Loda, M. Sc.

Department of Psychosomatic Medicine and Psychotherapy, University Hospital Tuebingen, Osianderstr. 5, D-72076 Tuebingen, Germany

Phone: 0049 7071 29 73688

Fax: 0049 7071 294382

e-mail: teresa.loda@med.uni-tuebingen.de

For peer review only - http://bmjopen.bmj.com/site/about/guidelines.xhtml

Word count: 2794 words

ABSTRACT

Objectives

Restrictions due to the COVID-19 pandemic mandated fundamental changes to student evaluations, including the administration of the Observed Structured Clinical Exam (OSCE). This study aims to conduct an in-person OSCE to verify students' practical skills under necessary infection-control practices and the impact of face masks on student–patient interactions.

Design

Cross-sectional design.

Setting

The OSCE at Medical School of Tuebingen takes place in October 2020.

Participants

A total of 149 students (third year of study) completed the survey (RR = 80.1%). It was their first OSCE.

Primary and Secondary outcome measures

Primary outcome measure was how this type of OSCE was evaluated by participating students in regard to preparation, content and difficulty as well as in real life. Secondary outcome measures were how the implemented hygiene actions influenced the OSCE, including the interaction and communication between students and SPs. Items were rated on a six-point Likert scale (1 = completely - 6 = not at all). Means, standard deviations, frequencies and percentages were calculated.

Results

For peer review only

149 students, 32 SPs and 59 examiners participated. The students rated the OSCE with 2.37 (±0.52) for preparation and 2.07 (±0.32) for content. They perceived the interaction to be significantly disrupted by the use of face masks (3.03 ± 1.54) ($p < .001$) compared to the SPs (3.84±1.44) and the examiners (4.14 ± 1.55). In general, the three groups considered the use of face masking the OSCE to be helpful (1.60 ± 1.15).

Conclusions

An in-person OSCE, even in the midst of a global pandemic, is feasible and acceptable to both students and faculty. When compared the students' results to previous students' results who completed the OSCE before the pandemic, the results indicated that students felt less prepared than under non-pandemic circumstances, however, their performances on this OSCE was not lower.

Strengths and limitations of this study

- The study presents the evaluation of an in-person OSCE in the height of this pandemic
- Best to our knowledge, this is the first investigation of the influence of face masks regarding communication and interaction in an OSCE
- The perspective of all people involved in the OSCE were assessed.
- One limitation is that the students might feel more stressed during the OSCE as it is an examination
- The study was conducted at only one medical faculty

Key words: OSCE, COVID-19, undergraduate medical students, face mask.

For peer review only - http://bmjopen.bmj.com/site/about/guidelines.xhtml

INTRODUCTION

The COVID-19 pandemic resulted in significant adaptations for teaching in medical schools, with a forced emphasis on digitalisation; for example, e-learning platforms, virtual training and video-conferencing have become established strategies for teaching students and others studying in the medical field.[1-4] Initially, traditional face-to-face teaching formats such as physical examinations had to be paused completely due to stringent governmental orders and the need to develop appropriate infection-control practices.[5 6] This change in format had impacts on not only how to teach but also what to teach, as some clinical skills, in particular communication skills training, are difficult to adapt from in-person to remote sessions. However, telemedicine has become a valuable option for teaching such clinical and communication skills.[7]

Despite new opportunities, this alternative method of teaching remains challenging, particularly the question of how to perform corresponding, valid exams under restrictions required to prevent the spread of COVID-19. Various approaches had to be tried to accommodate different phases of the pandemic itself.[1 8] Catalogues of learning objectives also had to be adapted accordingly, resulting in direct impacts on the content of various exams. With great effort, exams themselves had to be altered, either reformulated as online versions with all the related data-protection implications or held in classrooms under strict hygienic measures. Clinical and communication skills examinations, in particular, were a highly significant challenge and were sometimes omitted entirely during the first COVID-19 semester in summer 2020 as the risk of infection was too high. This especially affected the creation and administration of observed, structured clinical examinations (OSCEs) with their interdisciplinary setting evaluated by teachers of various disciplines.[9] The OSCE represents a standard assessment tool for medical training and is considered the most reliable and valid clinical examination system in undergraduate training[10]; therefore, a way to implement the OSCE during the pandemic COVID-19 had to be identified. Several universities solved this problem by conducting virtual or online OSCEs using Zoom video-conferencing.[11-15] However, the degree of realism in the virtual OSCE is limited, and the OSCE's organizational team needed to be prepared to manage and resolve technical difficulties.[11]

For peer review only - http://bmjopen.bmj.com/site/about/guidelines.xhtml

When the curve of the COVID-19 pandemic flattened, the Medical Faculty of Tuebingen decided that courses including clinical examinations should go back to being held in person, maintaining appropriate and strict infection-control practices since such clinical encounters represent an essential component of medical training. This particularly affected the OSCE as the faculty's central interdisciplinary, clinical examination. In this context, appropriate hygiene measures were developed by the OSCE coordination team based on the university and government specifications in order to minimize infection risk for all persons involved in the OSCE including students, examiners, and SPs. Here, we describe not only our successes in this effort but also several of the challenges we faced.

Aim

This study aims to present one method for facilitating an OSCE during a real-world pandemic situation. Thus, as primary aim, this study investigated how this type of OSCE was evaluated by participating students in regard to preparation, content and difficulty as well as in real life. Furthermore, as secondary aim, we investigated how the implemented hygiene measures influenced the OSCE, including the interaction and communication between students and SPs.

METHODS

Study design

This study presents a cross-sectional study at the University of Tuebingen.

The OSCE at the University of Tuebingen before the COVID-19 pandemic

The OSCE at the Medical School of Tuebingen takes place at the end of the third year of study. Nine clinical subjects participate in the OSCE: dermatology, general medicine, internal medicine, neurology, orthopaedics, paediatrics, psychosomatic medicine, radiology and surgery. The aim of the OSCE is the examination of students' clinical and communication skills. Clinical skills were represented by performing a clinical examination or by interpreting a medical report. Communication skills were assessed by the way the students interacted with the patients, e.g. by asking the patient for his or her name or introducing themselves using their name and function or by how they structured the communication with patients. Typically, the students are examined at 16 exam stations representing basic tasks such as

For peer review only - http://bmjopen.bmj.com/site/about/guidelines.xhtml

taking a patient's medical history, performing a clinical examination or interpreting medical findings (e.g. an x-ray). The task at each station lasts six minutes with a one-minute break to change places. All interactive stations (medical history, physical examination) include standardized patients (SPs) to provide a more realistic experience. Students are evaluated by discipline-specific examiners using an electronic standardised checklist consisting of 25 items on a tablet. Usually, about 180 medical students take part in the OSCE per semester.

Implementation of the OSCE at the University of Tuebingen during the COVID-19 pandemic

During the pandemic, OSCEs took place in October 2020. The 7-day incidence rate at this time was 88 per 100,000 (https://www.tagblatt.de/Nachrichten/Live-Blog, 11.05.21). Rapid testing had not yet been established as a means to test all participants for the coronavirus. Thus, the challenge was to adapt the OSCE to the university's hygiene measures including maintaining a social distance of at least 1.5 meters during the OSCE and limiting the number of persons involved. As the number of participating students per run was limited to 16, the OSCE was extended to three instead of two days, including six thematic blocks. The general structure of nine clinical subjects and 16 exam stations was retained. In addition, the exam time was kept to six minutes per station. However, the break-time between stations was doubled to two minutes to accommodate performing hygienic procedures (e.g. wiping all surfaces). All persons involved in the OSCE, including students, examiners, SPs and the coordination team, were required to wear face masks (minimum standard FFP1) for the total duration of the OSCE. As the OSCE always mirrors the clinical condition participants were not wearing disposable aprons. This also meant that several established stations had to be adapted because, as a result, several procedures were not possible (e.g. examination of the larynx, facial nerve testing). For physical examinations, students were required to wear surgical gloves, dispose of them after each examination, and put on a new pair for each new SP they met.

Evaluation

The evaluation of the OSCE during the COVID-19 pandemic consisted of two parts: (1) a general evaluation (established standard) and (2) a COVID-19 pandemic-specific evaluation (resulting from changes due to COVID-19).

For peer review only - http://bmjopen.bmj.com/site/about/guidelines.xhtml

General evaluation

The general evaluation included questions on the organization of the OSCE as well as rating the preparation, content and difficulty of each exam station. The students were also asked if they felt confident in their ability to perform each examined task in a real-word situation. Finally, students were given the opportunity to comment on the OSCE in a free-text section. All items were assessed by academic grades (1–6).

The COVID-19 pandemic-specific evaluation

The specific evaluation in relation to COVID-19 referred to the effects of wearing face masks and was divided into the following topics: *interaction, non-verbal communication, face mask in general,* and *face mask during OSCE*. An example of an item for *interaction* was 'The face mask influenced the interaction with the SP'. *Non-verbal communication* was assessed by the item 'They [students] made more use of the eyes in order to communicate with the SP'. An example of an item for *face mask in general* was represented by 'In general, I consider wearing a face mask as reasonable'. The topic *face mask during OSCE* was measured by items such as: 'Wearing a face mask affects taking the medical history/performing a physical examination'. All items were rated on a six-point Likert scale ranging from 1 ('completely') to 6 ('not at all').

Procedure

The participating students first completed the OSCE and then completed the general and COVID-19-specific evaluations. In addition, SPs and examiners completed the general evaluation after the OSCE. Both groups rated the students' clinical skills, communication skills and empathy after completing each student's checklist in the two-minute-break window.

Ethics

The study received ethics approval from the Ethics Committee of Tuebingen Medical Faculty (no. 314/2020BO2). Participation was voluntary, and students were not reimbursed or rewarded in any way for participating. All participants provided their written informed consent, and all of their responses and data were kept anonymous.

For peer review only - http://bmjopen.bmj.com/site/about/guidelines.xhtml

For peer review only

Patient and Public Involvement

Patients or the public were not involved in the design, or conduct, or reporting, or dissemination plans of our research.

Data analysis

A sample size of 90 students was needed for analysis by using the standard statistical formula for sample size including study population = 180, level of significance = .95, and margin of error = .5. The normal distribution of the data was confirmed by using the Kolmogorov–Smirnov test. Descriptive data, including mean values *(M),* standard deviations *(SD),* frequencies and percentages of relevant factors, were calculated. Any missing value was replaced with the mean value. The overall mean of missing values was estimated as 1.34%. Missing values were considered only if at least 80% of each item of the questionnaire had been completed. Using the Little missing completely at random test, it was confirmed that the data were missing randomly. The expectation-maximization algorithm was used to input the missing data.[16] *T* test for independent samples and ANOVAs were conducted to compare the results between the three groups including students, SPs and examiners. When data were not normally distributed, the Kruskal-Wallis test was performed to compare results. Bonferroni correction was used to correct multiple testing.[17] The Statistical Package for the Social Sciences version 26.0 (IBM, Armonk, NY, USA) was used for data analysis. The level of significance was set at $p < .05$.

RESULTS

Demographics

A total of 149 students completed the examination as well as the survey (RR = 80.1%). Also participating in the survey were 32 standardized patients (RR = 91.4%) and 59 examiners (RR = 92.2%). All medical students were in their third year of study.

General evaluation

For peer review only - http://bmjopen.bmj.com/site/about/guidelines.xhtml

Students evaluated the OSCE in regard to preparation at 2.37 (±0.52) and content and difficulty at 2.07 (±0.32). The confidence to perform the tasks in a real-world setting was rated 2.36 (±0.47). Both SPs and examiners rated the students' clinical skills as good (SPs: 1.83±0.79; examiners: 1.81±0.72, p > .05). Similar results were found for students' communication skills (SPs: 1.72±0.77; examiners: 1.70±0.57, p > .05). In regard to students' empathy with SPs, there was a significant effect (p < .05) showing a higher rating by the examiners (1.51±0.55) when compared to the SPs (1.79±0.78).

COVID-19-specific evaluation

Interaction and non-verbal communication

In regard to interaction and non-verbal communication, the results showed significant differences (p < .001) for the three groups, i.e. students, SPs and examiners (see Table 1 and Figure 1). Students rated the single items of interaction and non-verbal communication with higher agreement than did the SPs and examiners. The students were significantly in greater agreement that wearing a face mask could lead to misunderstandings in comparison to the SPs and examiners, who both disagreed with the statement. Overall, compared to students and examiners, the SPs were in significant disagreement that face masks affected non-verbal communication by, e.g. speaking more loudly or using more gestures.

Table 1. *Interaction and non-verbal communication.*

**Items***	**Students**		**SPs**		**Examiners**		**Statistics**
	M	SD	M	SD	M	SD	p-value
In general, interaction is affected by face masks.	3.03	1.54	3.84	1.44	4.14	1.55	< .001
Face masks have a negative impact on specific interactions with SPs.	2.92	1.58	3.59	1.58	3.85	1.68	< .001
Face masks lead to misunderstandings with SPs.	3.85	1.59	5.43	1.04	4.82	1.38	< .001

Figure 1 about here

For peer review only - http://bmjopen.bmj.com/site/about/guidelines.xhtml

The use of face masks in general and in the OSCE

Students, SPs and examiners disagreed that wearing face masks had a negative impact on their well-being (range: 4.19–4.91). They all rated the face mask as useful in the OSCE (range: 1.52–1.79). However, students were more likely to agree that they would have preferred the option of participating in the OSCE the following semester without wearing a face mask (3.03±1.84). In regard to the content of the examination, SPs and examiners significantly disagreed that wearing a mask interfered with conducting an anamnesis. Furthermore, the SPs significantly disagreed that wearing a face mask interfered with performing a physical examination during the OSCE. In addition, compared to SPs and examiners, students significantly disagreed that face masks affected communication during the OSCE. SPs and examiners significantly disagreed that face masks have negative impact on the students' grade. Please see Table 2 and Figure 2 for additional details.

Table 2. *The use of face masks in general and in the OSCE.*

**Items***	**Students**		**SP**		**Examiner**		**Statistics**
	M	SD	M	SD	M	SD	p-value
Face masks, in general, are useful.	1.58	1.08	2.67	2.13	1.92	1.34	< .001
A face mask has a negative impact on my well-being.	4.19	1.76	4.91	1.35	4.54	1.56	> .05
Wearing a face mask causes physical symptoms (e.g. headaches, getting less air, etc.).	4.68	1.63	5.47	0.95	4.79	1.68	< .05
Wearing a face mask interfered with conducting the anamnesis.	3.68	1.73	4.70	1.34	4.76	1.56	< .001
Wearing a face mask interfered with performing a physical examination during the OSCE.	3.81	1.65	5.33	1.04	4.44	1.80	< .001
Face masks have a negative impact on the academic grade.	4.03	1.64	4.93	1.86	5.00	1.58	< .001

For peer review only - http://bmjopen.bmj.com/site/about/guidelines.xhtml

*Items presented are from the students' questionnaire version; SPs/examiners had respective corresponding items (e.g. 'Wearing face masks interfered with the students conducting the anamnesis'.

Figure 2 about here

DISCUSSION

This study investigated the implementation of an OSCE during a pandemic. Regarding students' clinical and communication skills, SPs and examiners rated them as good, while the students themselves felt less confident about performing the required tasks in a real-world setting. When focussing on the changes required for appropriate hygienic measures, the SPs reported that face masks affected non-verbal communication, including speaking louder or using more eye contact, less in comparison to the examiners and students. Students, however, rated the face masks as having influenced the interaction and non-verbal communication with the SPs; therefore, they used more eye contact and spoke louder. In general, all participants found face masks to be a useful tool in the OSCE during the pandemic and reported that the masks did not interfere with performing the OSCE in general.

Evaluation of an OSCE during a pandemic

When compared to previous results, i.e. OSCE before COVID-19, the results indicated that students felt less prepared for this OSCE.[9] They also evaluated the content as more challenging and the difficulty as greater for the OSCE under the conditions required due to the pandemic.[9] Furthermore, they felt less confident about performing tasks such as taking a medical history or conducting a physical examination in a real-world setting, although the SPs and examiners rated their clinical and communications skills as good. The SPs' and examiners ratings of clinical and communication skills were similar to those of students' exam performances on previous OSCEs.[18 19] The reason may be that problematic circumstances during the semester (digital teaching, less emphasis on clinical skills) seem to have been counterbalanced by more-lenient examiners who kept these difficulties in mind.

Furthermore, the OSCE presents a stressful exam situation in which many students feel distressed and anxious regardless of unusual circumstances such as those caused by the

For peer review only - http://bmjopen.bmj.com/site/about/guidelines.xhtml

COVID-19 pandemic.[9 20 21] This year, the students experienced even greater distress during the OSCE since the academic courses to prepare them for it were transferred to online formats.[22] Thus, students might feel less confident about performing the tasks in a real-world situation.

Face masks and interaction

In regard to interaction and non-verbal interaction, students were afraid that wearing face masks might affect their interaction with the SPs and that non-verbal communication might interfere with the SP during the tasks. Similar fears were also reported by psychiatrists who emphasised that the face masks contribute to a psycho-social barrier in the patient–physician interaction.[23] In addition, students agreed to speak louder when wearing a face mask. This phenomenon was confirmed by a previous study where a speech intelligibility assessment of protective face masks and air-purifying respirators was investigated.[24] However, examiners and SPs perceived the face masks as less disruptive than the students did. Examiners as clinicians may have become accustomed to them. Furthermore, SPs were specifically instructed in advance about how to communicate while wearing a face mask. Finally, all participants agreed that wearing face masks did not lead to any misunderstandings with the SPs, which was in contrast to previous studies that investigated face masks in relation to patient–physician communication.[25]

Face masks in the OSCE

Wearing face masks was seen as useful in general, as well as in the OSCE exam, by students, SPs and examiners. This finding aligns with those of previous investigations of face masks.[25-27] Moreover, all participants disagreed that face masks might interfere with performing a physical examination or taking a medical history during the OSCE. While Campagne [28] emphasised that face masks contribute to distress in patient communication, the students in the present study reported that they did not affect the communication in the OSCE and that they were able to structure and take charge of the conversation despite face masks.[28] SPs and examiners agreed with the students. Medical students are well-informed about COVID-19 and are aware of its consequences in academic and healthcare contexts.[22 29] Thus, the students might have felt confident about conducting a conversation while wearing a face mask in the OSCE. All participants also agreed that wearing face masks had no negative impact on the students' grades in the OSCE.

For peer review only - http://bmjopen.bmj.com/site/about/guidelines.xhtml

For peer review only

Strengths and limitations

Based on the results, a strength of this study includes the finding that an in-person OSCE can be effectively performed even during a severe pandemic if it is done in a thoughtful and structured format, keeping both the educational objectives as well as proper infection-control practices in mind. Moreover, best to our knowledge, the study is the first investigation of the influence of face masks regarding communication and interaction in an OSCE. Furthermore, the perspective of all people involved in the OSCE were assessed.

A limitation of this study is the higher level of stress in medical students during the OSCE as it is an examination and stress has complex interactions with students' performance and the way how they judge situations. [30]Additionally, the study was conducted at only one medical faculty; thus, the results cannot be generalized. Furthermore, the cross-sectional study design only allows to capture the status of the current situation and a longitudinal study design is needed to determine possible causal effects. Despite its limitations, we believe that this study contributes to the knowledge about how to conduct effective OSCEs and possibly other student assessments in unusual circumstances such as the COVID-19 pandemic, as well as the challenges that must be considered.

Conclusion

Wearing face masks did not interfere with student performance regarding clinical and communication skills in the OSCE exam. Students felt less prepared for the OSCE as 'refresher' courses were missing before the OSCE took place, and academic courses were transferred to online formats where relevant teaching elements, e.g. of physical examination, could be lost.[22] Again, these results showed that students were aware of the circumstances and consequences of COVID-19. In conclusion, an in-person OSCE, even in the midst of a global pandemic, is feasible and acceptable to both students and faculty. Furthermore, students are willing to fulfil the hygienic measurements like wearing a face mask. However, the clinical educators are prompted to create and implement 'refresher' courses in order to deliver good preparation for students and to compensate the missing face-to-face courses before the OSCE. Future research might also focus on the students' grades and performances in the OSCE during the pandemic, including a comparison with pre-pandemic OSCE results before the restrictions and changes required to prevent the spread of COVID-19.[9]

For peer review only - http://bmjopen.bmj.com/site/about/guidelines.xhtml

Acknowledgements
We thank Lea Herschbach for her support as study assistant.

Declaration of interest statement
The authors declare that they have no competing interests.

Funding/Support
We acknowledge support with financing publication fees by 'Deutsche Forschungsgemeinschaft' and Open Access Publishing Fund of the University of Tuebingen (both grant numbers are not applicable).

Data availability statement
Complete data are available on reasonable request to the corresponding author.

Notes on contributors

Teresa Loda, Dr, M Sc, is a psychologist and research scientist at the Department for Psychosomatic Medicine and Psychotherapy at University Hospital Tübingen.

Andrew B. Nevins, Assitant Prof, MD, is a clinical assistant professor of medicine (infectious diseases) at the Standford University School of Medicine.

Rebecca Erschens, Dr, M Sc is a clinical psychologist, research scientist and lecturer at the Department for Psychosomatic Medicine and Psychotherapy at University Medical Hospital Tuebingen.

Stephan Zipfel Prof., MD., is Professor for Psychosomatic Medicine and Psychotherapy at University Hospital Tuebingen, and dean for student affairs at the Faculty of Medicine of Tuebingen University.

Anne Herrmann-Werner, Prof, MD, MME, a physician for psychosomatic medicine and psychotherapy by training, is full professor for Medical Education at the University Hospital and Medical Faculty Tübingen.

Author's contribution
AHW and TL were responsible for the design and conduction the study, as well as acquisition, analysis and interpretation of data. AHW and TL drafted the first version of the manuscript. RE and AN was involved in data analyses and interpretation and revised the manuscript critically. SZ made substantial contributions to the study design and revised the

For peer review only - http://bmjopen.bmj.com/site/about/guidelines.xhtml

manuscript critically. All authors approved the final version of the manuscript and agreed to be accountable for all aspects of the work.

Figure caption

Figure 1. Face mask and non-verbal communication in patient–physician communication. Items presented are from the students' questionnaire version; SPs/examiners had respective corresponding items (e.g. 'the student spoke louder'). All items were rated on a six-point Likert scale ranging from 1 ('completely') to 6 ('not at all').

Figure 2. Face mask and patient-physician communication in the OSCE. Items presented are from the students' questionnaire version; SPs/examiners had respective corresponding items (e.g. 'the student could structure the conversation despite face mask'). All items were rated on a six-point Likert scale from ranging from 1 ('completely') to 6 ('not at all').

References

1. Rose S. Medical Student Education in the Time of COVID-19. *Jama* 2020;323(21):2131-32. doi: 10.1001/jama.2020.5227 [published Online First: 2020/04/02]
2. Liang ZC, Ooi SBS, Wang W. Pandemics and Their Impact on Medical Training: Lessons From Singapore. *Academic medicine : journal of the Association of American Medical Colleges* 2020;95(9):1359-61. doi: 10.1097/ACM.0000000000003441
3. Evans DJ, Bay BH, Wilson TD, et al. Going virtual to support anatomy education: A STOPGAP in the midst of the Covid-19 pandemic: Wiley Online Library, 2020.
4. Sandars J, Correia R, Dankbaar M, et al. Twelve tips for rapidly migrating to online learning during the COVID-19 pandemic. *MedEdPublish* 2020;9
5. Whelan A PJ, Young G, Catanese V, McKinney R. . Interim guidance on medical students' participation in direct patient contact activities: principles and guidelines. *Association of American Medical Colleges* 2020
6. Whelan A PJ, Young. Guidance on medical stu-dents' clinical participation: effective immediatel. *Academic Medicine* 2020
7. Iancu AM, Kemp MT, Alam HB. Unmuting Medical Students' Education: Utilizing Telemedicine During the COVID-19 Pandemic and Beyond. *J Med Internet Res* 2020;22(7):e19667. doi: 10.2196/19667
8. Theoret C, Ming X. Our education, our concerns: The impact on medical student education of COVID-19. *Medical education* 2020;54(7):591-92. doi: 10.1111/medu.14181 [published Online First: 2020/05/23]
9. Graf J, Smolka R, Holderried F, et al. Ten years of objective structured clinical examination at the medical Faculty of Tübingen, Germany: item analysis and students' satisfaction. *J Health Sci Educ* 2018;2(2):133.
10. Walters K, Osborn D, Raven P. The development, validity and reliability of a multimodality objective structured clinical examination in psychiatry. *Medical Education* 2005;39(3):292-98. doi: https://doi.org/10.1111/j.1365-2929.2005.02091.x
11. Khan KZ, Ramachandran S, Gaunt K, et al. The Objective Structured Clinical Examination (OSCE): AMEE Guide No. 81. Part I: an historical and theoretical perspective. *Med Teach* 2013;35(9):e1437-46. doi: 10.3109/0142159x.2013.818634 [published Online First: 2013/08/24]

For peer review only - http://bmjopen.bmj.com/site/about/guidelines.xhtml

12. Kakadia R, Chen E, Ohyama H. Implementing an online OSCE during the COVID-19 pandemic. *Journal of dental education* 2020
13. Donn J, Scott JA, Binnie V, et al. A pilot of a Virtual Objective Structured Clinical Examination in dental education. A response to COVID-19. *European Journal of Dental Education* 2020
14. Major S, Sawan L, Vognsen J, et al. COVID-19 pandemic prompts the development of a Web-OSCE using Zoom teleconferencing to resume medical students' clinical skills training at Weill Cornell Medicine-Qatar. *BMJ Simulation & Technology Enhanced Learning* 2020
15. Medical Faculty's and Students' Perceptions toward Pediatric Electronic OSCE during the COVID-19 Pandemic in Saudi Arabia. Healthcare; 2021. Multidisciplinary Digital Publishing Institute.
16. Musil CM, Warner CB, Yobas PK, et al. A comparison of imputation techniques for handling missing data. *Western Journal of Nursing Research* 2002;24(7):815-29.
17. Fahrmeir L, Heumann C, Künstler R, et al. Statistik: Der weg zur datenanalyse: Springer-Verlag 2016.
18. Graf J, Loda T, Zipfel S, et al. Communication skills of medical students: survey of self- and external perception in a longitudinally based trend study. *BMC Medical Education* 2020;20(1):149. doi: 10.1186/s12909-020-02049-w
19. Graf J, Smolka R, Simoes E, et al. Communication skills of medical students during the OSCE: Gender-specific differences in a longitudinal trend study. *BMC Medical Education* 2017;17(1):75. doi: 10.1186/s12909-017-0913-4
20. Brand HS, Schoonheim-Klein M. Is the OSCE more stressful? Examination anxiety and its consequences in different assessment methods in dental education. *European Journal of Dental Education* 2009;13(3):147-53. doi: https://doi.org/10.1111/j.1600-0579.2008.00554.x
21. Gilani S, Pankhania K, Aruketty M, et al. Twelve tips to organise a mock OSCE. *Medical Teacher* 2021:1-20. doi: 10.1080/0142159X.2021.1887465
22. Loda T, Löffler T, Erschens R, et al. Medical education in times of COVID-19: German students' expectations – A cross-sectional study. *PLOS ONE* 2020;15(11):e0241660. doi: 10.1371/journal.pone.0241660
23. Padhy SK, Rina K, Sarkar S. Smile, grimace or grin? Recalibrating psychiatrist-patient interaction in the era of face masks. *Asian journal of psychiatry* 2020;53:102389-89. doi: 10.1016/j.ajp.2020.102389 [published Online First: 2020/08/27]
24. Palmiero AJ, Symons D, Morgan JW, et al. Speech intelligibility assessment of protective facemasks and air-purifying respirators. *Journal of Occupational and Environmental Hygiene* 2016;13(12):960-68. doi: 10.1080/15459624.2016.1200723
25. Marler H, Ditton A. "I'm smiling back at you": Exploring the impact of mask wearing on communication in healthcare. *International Journal of Language & Communication Disorders* 2021;56(1):205-14. doi: https://doi.org/10.1111/1460-6984.12578
26. Spitzer M. Masked education? The benefits and burdens of wearing face masks in schools during the current Corona pandemic. *Trends in Neuroscience and Education* 2020;20:100138. doi: https://doi.org/10.1016/j.tine.2020.100138
27. Bakhit M, Krzyzaniak N, Scott AM, et al. Downsides of face masks and possible mitigation strategies: a systematic review and meta-analysis. *BMJ Open* 2021;11(2):e044364. doi: 10.1136/bmjopen-2020-044364
28. Campagne DM. The problem with communication stress from face masks. *Journal of Affective Disorders Reports* 2021;3:100069. doi: https://doi.org/10.1016/j.jadr.2020.100069
29. Taghrir MH, Borazjani R, Shiraly R. COVID-19 and Iranian Medical Students; A Survey on Their Related-Knowledge, Preventive Behaviors and Risk Perception. *Arch Iran Med* 2020;23(4):249-54. doi: 10.34172/aim.2020.06
30. Yerkes RM, Dodson JD. The relation of strength of stimulus to rapidity of habit-formation. 1908

For peer review only

For peer review only - http://bmjopen.bmj.com/site/about/guidelines.xhtml

For peer review only

For peer review only - http://bmjopen.bmj.com/site/about/guidelines.xhtml

Figure 1

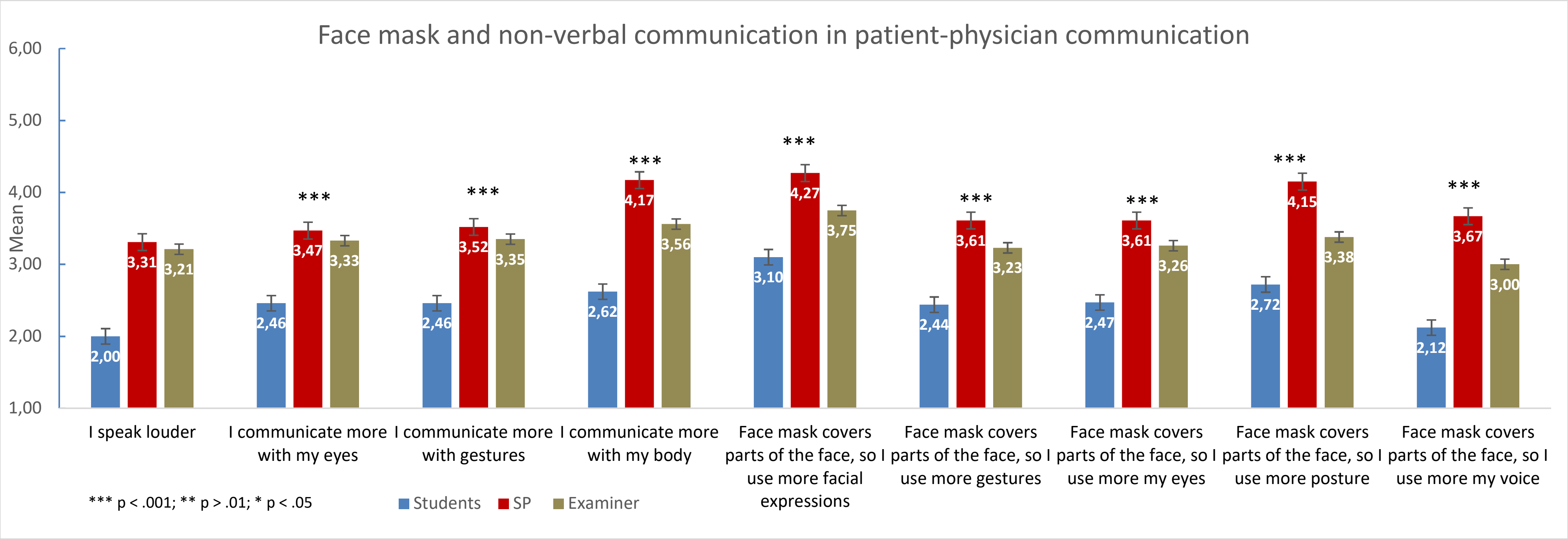
Face mask and non-verbal communication in patient-physician communication
Mean
6,00
5,00
4,00
3,00
2,00
1,00
2,00
3,31
3,21
***
2,46
3,47
3,33
***
2,46
3,52
3,35
***
2,62
4,17
3,56
***
3,10
4,27
3,75
***
2,44
3,61
3,23
***
2,47
3,61
3,26
***
2,72
4,15
3,38
***
2,12
3,67
3,00
I speak louder
I communicate more with my eyes
I communicate more with gestures
I communicate more with my body
Face mask covers parts of the face, so I use more facial expressions
Face mask covers parts of the face, so I use more gestures
Face mask covers parts of the face, so I use more my eyes
Face mask covers parts of the face, so I use more posture
Face mask covers parts of the face, so I use more my voice
*** p < .001; ** p > .01; * p < .05
Students
SP
Examiner

Figure 2

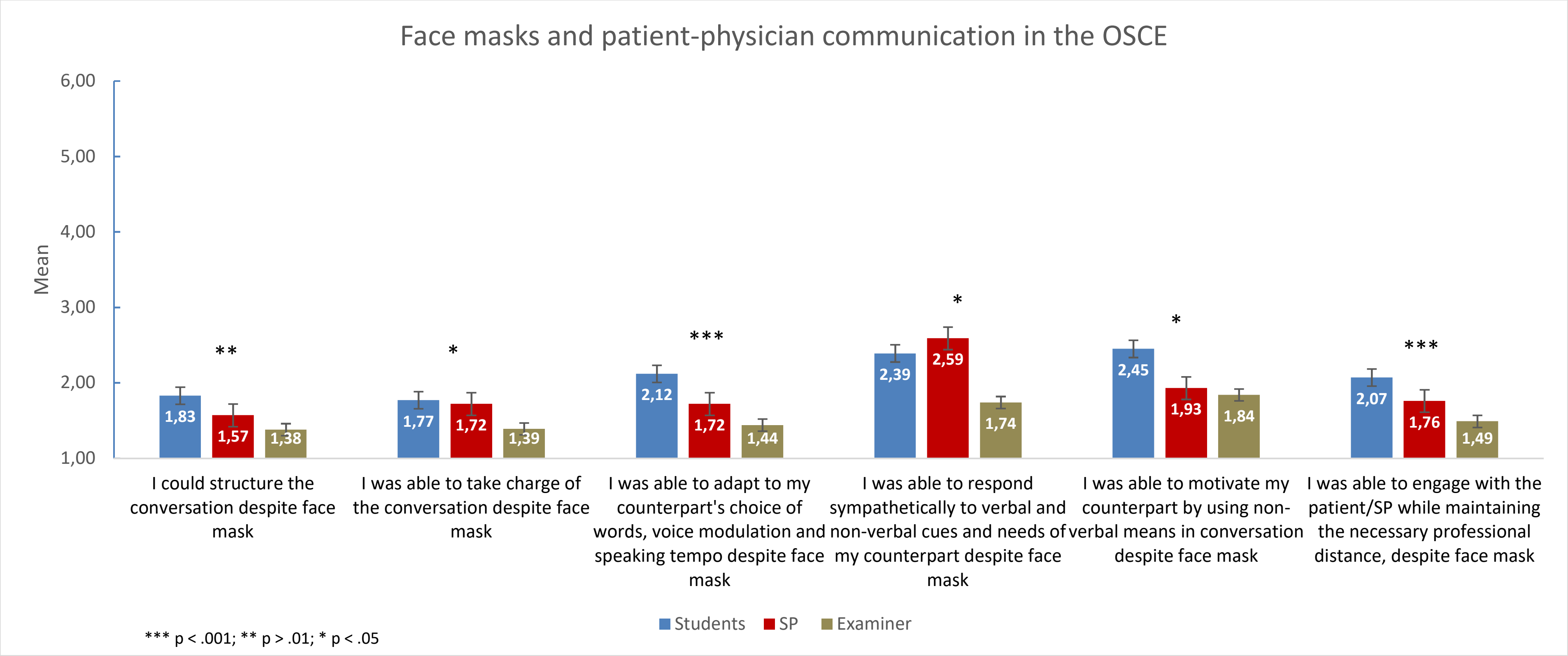
Face masks and patient-physician communication in the OSCE
Mean
6,00
5,00
4,00
3,00
2,00
1,00
1,83
1,57
1,38
1,77
1,72
1,39
2,12
1,72
1,44
2,39
2,59
1,74
2,45
1,93
1,84
2,07
1,76
1,49
**
*
***
*
*
***
I could structure the conversation despite face mask
I was able to take charge of the conversation despite face mask
I was able to adapt to my counterpart's choice of words, voice modulation and speaking tempo despite face mask
I was able to respond sympathetically to verbal and non-verbal cues and needs of my counterpart despite face mask
I was able to motivate my counterpart by using non-verbal means in conversation despite face mask
I was able to engage with the patient/SP while maintaining the necessary professional distance, despite face mask
Students
SP
Examiner
*** p < .001; ** p > .01; * p < .05

For peer review only

STROBE 2007 (v4) Statement—Checklist of items that should be included in reports of *cross-sectional studies*

Section/Topic	Item #	Recommendation	Reported on page #
**Title and abstract**	1	(*a*) Indicate the study's design with a commonly used term in the title or the abstract	1
		(*b*) Provide in the abstract an informative and balanced summary of what was done and what was found	2-3
**Introduction**			**4-6**
Background/rationale	2	Explain the scientific background and rationale for the investigation being reported	4-5
Objectives	3	State specific objectives, including any prespecified hypotheses	5
**Methods**			**5-8**
Study design	4	Present key elements of study design early in the paper	5
Setting	5	Describe the setting, locations, and relevant dates, including periods of recruitment, exposure, follow-up, and data collection	6
Participants	6	(*a*) Give the eligibility criteria, and the sources and methods of selection of participants	5-6
Variables	7	Clearly define all outcomes, exposures, predictors, potential confounders, and effect modifiers. Give diagnostic criteria, if applicable	6-7
Data sources/ measurement	8*	For each variable of interest, give sources of data and details of methods of assessment (measurement). Describe comparability of assessment methods if there is more than one group	6-7
Bias	9	Describe any efforts to address potential sources of bias	*N.A*
Study size	10	Explain how the study size was arrived at	8
Quantitative variables	11	Explain how quantitative variables were handled in the analyses. If applicable, describe which groupings were chosen and why	8
Statistical methods	12	(*a*) Describe all statistical methods, including those used to control for confounding	8
		(*b*) Describe any methods used to examine subgroups and interactions	8
		(*c*) Explain how missing data were addressed	8
		(*d*) If applicable, describe analytical methods taking account of sampling strategy	n.a.
		(*e*) Describe any sensitivity analyses	n.a.
**Results**			8-10

For peer review only

Participants	13*	(a) Report numbers of individuals at each stage of study—eg numbers potentially eligible, examined for eligibility, confirmed eligible, included in the study, completing follow-up, and analysed	8
		(b) Give reasons for non-participation at each stage	n.a.
		(c) Consider use of a flow diagram	n.a.
Descriptive data	14*	(a) Give characteristics of study participants (eg demographic, clinical, social) and information on exposures and potential confounders	8
		(b) Indicate number of participants with missing data for each variable of interest	8
Outcome data	15*	Report numbers of outcome events or summary measures	8-9
Main results	16	(*a*) Give unadjusted estimates and, if applicable, confounder-adjusted estimates and their precision (eg, 95% confidence interval). Make clear which confounders were adjusted for and why they were included	9-10
		(*b*) Report category boundaries when continuous variables were categorized	9-10
		(*c*) If relevant, consider translating estimates of relative risk into absolute risk for a meaningful time period	9-10
Other analyses	17	Report other analyses done—eg analyses of subgroups and interactions, and sensitivity analyses	n.a.
**Discussion**			10-13
Key results	18	Summarise key results with reference to study objectives	10-12
Limitations	19	Discuss limitations of the study, taking into account sources of potential bias or imprecision. Discuss both direction and magnitude of any potential bias	12
Interpretation	20	Give a cautious overall interpretation of results considering objectives, limitations, multiplicity of analyses, results from similar studies, and other relevant evidence	11-13
Generalisability	21	Discuss the generalisability (external validity) of the study results	13
**Other information**			13
Funding	22	Give the source of funding and the role of the funders for the present study and, if applicable, for the original study on which the present article is based	13

*Give information separately for cases and controls in case-control studies and, if applicable, for exposed and unexposed groups in cohort and cross-sectional studies.

Note: An Explanation and Elaboration article discusses each checklist item and gives methodological background and published examples of transparent reporting. The STROBE checklist is best used in conjunction with this article (freely available on the Web sites of PLoS Medicine at http://www.plosmedicine.org/, Annals of Internal Medicine at http://www.annals.org/, and Epidemiology at http://www.epidem.com/). Information on the STROBE Initiative is available at www.strobe-statement.org.

For peer review only
